# Acute coronary syndrome associated cardiogenic shock in the catheterization laboratory: peripheral veno-arterial extracorporeal membrane oxygenator management and recommendations

**DOI:** 10.3389/fmed.2023.1277504

**Published:** 2023-11-07

**Authors:** Réka Ehrenberger, Balázs T. Németh, Péter Kulyassa, Gábor A. Fülöp, Dávid Becker, Boldizsár Kiss, Endre Zima, Béla Merkely, István F. Édes

**Affiliations:** Heart and Vascular Center, Semmelweis University, Budapest, Hungary

**Keywords:** cardiogenic shock (CS), percutaneous coronary intervention (PCI), acute coronary syndrome (ACS), extracorporeal life support (ECLS), veno-arterial extracorporeal membrane oxygenator (VA-ECMO)

## Abstract

Cardiogenic shock (CS) in acute coronary syndrome (ACS) is a critical disease with high mortality rates requiring complex treatment to maximize patient survival chances. Emergent coronary revascularization along with circulatory support are keys to saving lives. Mechanical circulatory support may be instigated in severe, yet still reversible instances. Of these, the peripheral veno-arterial extracorporeal membrane oxygenator (pVA-ECMO) is the most widely used system for both circulatory and respiratory support. The aim of our work is to provide a review of our current understanding of the pVA-ECMO when used in the catheterization laboratory in a CS ACS setting. We detail the workings of a Shock Team: pVA-ECMO specifics, circumstances, and timing of implantations and discuss possible complications. We place emphasis on how to select the appropriate patients for potential pVA-ECMO support and what characteristics and parameters need to be assessed. A detailed, stepwise implantation algorithm indicating crucial steps is also featured for practitioners in the catheter laboratory. To provide an overall aspect of pVA-ECMO use in CS ACS we further gave pointers including relevant human resource, infrastructure, and consumables management to build an effective Shock Team to treat CS ACS via the pVA-ECMO method.

## 1. Introduction

Cardiogenic shock (CS) is defined as generalized hypoperfusion and organ hypoxia due to primary cardiac dysfunction, which, if left untreated, leads to multiple organ failure and eventual death. A decrease in systolic blood pressure (<90 mmHg), and cardiac index (<2.2 l/min/m^2^), as well as an increase in pulmonary capillary wedge pressure (PCWP ≥ 15 mmHg) are parametrical values indicating the presence or onset of CS (1–4).

Acute coronary syndrome (ACS) presents as an emergency medical condition requiring urgent coronary revascularization, usually by means of percutaneous coronary intervention (PCI) performed at specialized cardiovascular centers. In cases of severe ACS, clinical manifestations of shock (CS ACS) may develop in 3–13% of patients due to acute left ventricular failure, significantly increasing the risk of mortality by over tenfold (1, 4–6).

**FIGURE 1:**
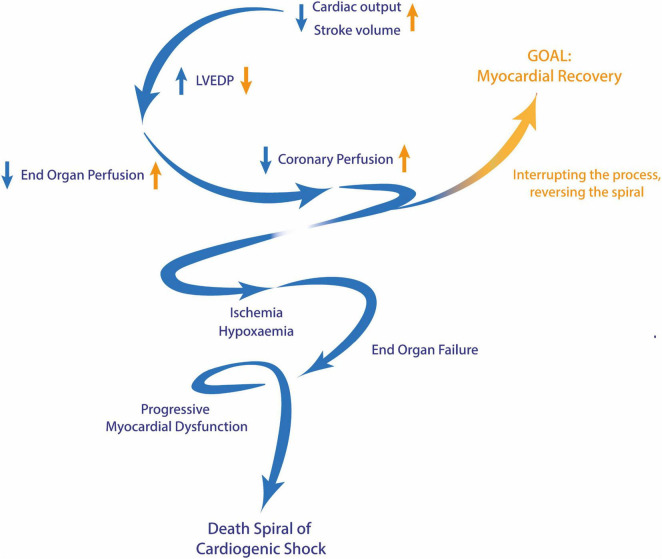
The death spiral of cardiogenic shock. Blue arrows represent the changes observed during the progression of cardiogenic shock, while yellow arrows depict the opposing changes achieved through effective and timely intervention. LVEDP, left ventricular end-diastolic pressure.

The pathophysiological progression toward CS, especially CS ACS is frequently described as a complex spiral, culminating in an irreversible state (4) (Figure 1). Consequently, the primary objective revolves around the timely identification of the condition prompting immediate and simultaneous treatment of the underlying ACS and circulatory failure in its early stages. In approximately half of CS ACS cases, comprehensive and accurate medical and interventional treatment can effectively halt the deterioration of the condition and provide a cure for the patients. However, if CS worsens and potentially or clinically proves refractory to lesser treatment methods, the utilization of extracorporeal life support (ECLS) devices becomes a viable option in dedicated cases (4, 7, 8).

The use of ECLS devices has been a longstanding practice in the field of cardiology, dating back to the 1960s (4). Among the array of available options, veno-arterial (VA) extracorporeal membrane oxygenator (ECMO) may be the optimal choice for cases of refractory cardiogenic shock, however, there is a notable absence of comprehensive guidelines concerning many aspects related to this device, particularly in terms of appropriate patient selection, timing and duration of VA-ECMO, and also the management of associated complications.

Our aim in this work is to offer insightful information regarding the utilization and management of peripheral VA-ECMO within the catheterization laboratory (cath lab) in cases of CS ACS, from the viewpoint of a high-volume tertiary Shock Center. Our objective is to present an overview of both scientific and empirical recommendations on this important topic.

## 2. Veno-arterial extracorporeal membrane oxygenator (VA-ECMO)

As a specific type of ECLS, VA-ECMO enables the temporary replacement of both respiratory and cardiovascular functions, thus providing support in acute, life-threatening clinical scenarios (9, 10). If initiated in properly selected patients in a timely manner, VA-ECMO can significantly mitigate the risk of multiple organ failure and eventual death (11, 12).

Veno-arterial extracorporeal membrane oxygenator supports cardiorespiratory function by providing continuous flow and oxygenation using an external capillary biomatrix. It concurrently maintains effective cardiac output and relieves pressure in the central veins, right atrium, and right ventricle, which ensures organ protection for the time required to evaluate the prognosis and allows for a decision to be made on the optimal therapeutic method (bridge to decision/bridge to bridge) (4, 10, 13). Furthermore, it serves as a viable option until either reversible damage is restored (bridge to recovery), or the implantation of a long-term mechanical circulatory support (MCS) device (destination therapy) becomes necessary. VA-ECMO treatment might ultimately be extended until heart transplantation (bridge to transplantation) becomes feasible (14, 15).

The newest data on cardiogenic shock-associated pVA-ECMO outcomes were recently published by Thiele et al. (16), as they randomized CS ACS early revascularization treatment to an ECLS-supported and a conservative (non-ECLS) arm. The study showed no 30-day mortality differences between the groups. However, interpreting these results requires understanding several limiting factors that the authors themselves emphasized. First, left ventricular unloading in the ECLS group was undertaken at a low percentage (5.8%). Additionally, severe bleeding complications reduced the potential benefits of the ECLS group, which however, may be mitigated or somewhat minimized by an experienced Shock Team. Most importantly, despite randomization crossover/bail-out ECLS was initiated in 12.5% of the control group, furthermore, other forms of MCS (other than ECMO, primarily a microaxial transvalvular device) were used in an additional 15.4% of the control patients. Thus, altogether, nearly one-third of the control group did eventually receive mechanical circulatory support in one form or another. These statistics underscore the importance of utilizing ECLS or other MCS devices in managing critically ill patients, especially those facing hemodynamic deterioration despite appropriate medical interventions.

Among these devices, the microaxial transvalvular device (Impella) is increasingly deployed in cases of CS ACS, either alongside or concurrently with VA-ECMO. Impella operates as a catheter-based, continuous axial flow pump, allowing for active propulsion of blood into the aorta, while reducing myocardial stress and enhancing systemic circulation (9, 17). The safety and efficacy of Impella and VA-ECMO in treating critically ill patients with CS ACS remains a topic of debate due to conflicting evidence. Most studies suggest little difference in mortality rates, although specific outcomes rates vary. Lemor et al. (18) found higher in-hospital mortality and complication rates in the ECMO group, while others reported no significant differences in short-term or long-term mortality (19–21). Given these discrepancies and the complex nature of CS ACS, ongoing research is crucial to guide device selection in clinical practice.

We believe, that within the context of acute coronary syndrome complicated by cardiogenic shock, the utilization of VA-ECMO remains fundamental. Nonetheless, its effectiveness depends on careful patient selection, accurate timing of insertion, and a keen awareness of the specific conditions of each case. By adopting a patient-focused strategy, the administration and management of VA-ECMO can be improved, potentially leading to better results in this critically ill patient population.

## 3. Circumstances and timing of implantation

The peripheral iteration of the system (pVA-ECMO) is a widely utilized therapeutic approach as a component of the complex management of refractory CS ACS (22, 23). The implantation is typically conducted in the cath lab or the intensive care unit of the cardiology department but may also be implemented beyond the confines of an in-hospital setting (24–27). It involves percutaneous cannulation of the major blood vessels of the inguinal (femoral artery and/or vein) or jugular (carotid artery and/or internal jugular vein) region with a large bore cannula for use as outflow and inflow sites of the pVA-ECMO (28, 29).

As per the recommendations of the European Society of Cardiology (14, 15), cardiovascular centers are encouraged to establish designated teams to define the most appropriate strategy for managing patients with cardiogenic shock. These Shock Teams should typically consist of a multidisciplinary group of experts specialized in cardiology, cardiothoracic surgery, critical care, and nursing, working collaboratively to provide optimal care for patients in such critical conditions (9, 15, 30).

### 3.1. Patient selection

Establishing selection criteria for appropriate candidates and determining the optimal timing for MCS is of paramount importance, especially considering that presently approximately 40–50% of CS ACS patients survive with standard medical therapy, while another 25–35% of cases may not respond to MCS effectively. The remaining 15–35% of patients with CS ACS, however, show clear benefits from MCS and eventually survive due to pVA-ECMO support (2, 4).

Several scoring systems have been developed to assess the risk classification of patients based on specific clinical and biological indicators. These scoring systems, such as the SAVE Score (31), ENCOURAGE (32), and PREDICT-VA ECMO (33), have the potential to enhance patient selection and optimize outcomes. The most prevalent SAVE Score (31) is based on a cohort analysis combining data from multiple international centers. This scoring system classifies patients into five distinct risk categories based on a comprehensive set of criteria pertaining to their medical history and pre-ECMO implantation condition. In terms of prognosis, it assigns hospital survival rates ranging from 75 to 18%. According to the findings of the study, primary contributors to higher mortality rates include pre-ECMO organ failure, chronic renal failure, an extended period of intubation before ECMO, pre-ECMO cardiac arrest, congenital heart disease, pulse pressure before ECMO ≤ 20 mmHg, and HCO_3_^–^ before ECMO ≤ 15 mmol/L. Conversely, factors that offer protection against increased mortality are younger age, lower body weight, and the underlying causes of cardiogenic shock being myocarditis, refractory VT/VF, or post-heart or lung transplantation, respectively.

**FIGURE 2:**
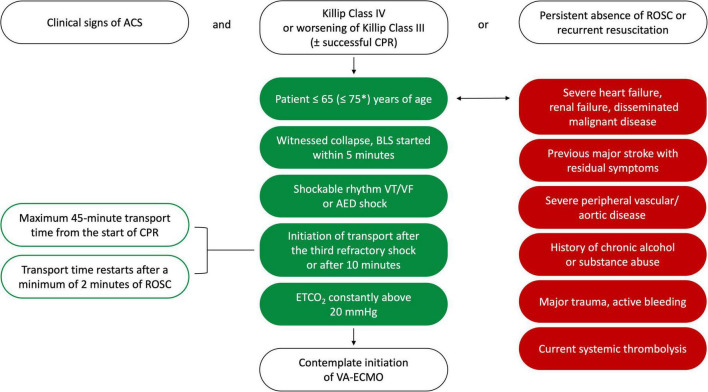
Protocol for peripheral VA-ECMO implantation in acute cardiogenic shock at the heart and vascular center. *If cardiac arrest occurs in a hospital setting, where the initiation of VA-ECMO is feasible within a 20-min timeframe. ACS, acute coronary syndrome; AED, automated external defibrillator; BLS, basic life support; CPR, cardiopulmonary resuscitation; ETCO_2_, end-tidal CO_2_; ROSC, return of spontaneous circulation; VA-ECMO, veno-arterial extracorporeal membrane oxygenator; VF, ventricular fibrillation; VT, ventricular tachycardia.

Nonetheless, in the absence of internationally accepted CS ACS pVA-ECMO initialization guidelines, our center has developed its own protocol (Figure 2), based on available literature data and empirical findings from our own clinical study using one of the largest single-center databases among the specialized institutions worldwide (34). The protocol considers multiple factors, such as clinical condition, age, low-flow times, and comorbidities. It also incorporates the recommendations of the European Society of Cardiology (14, 15) (IIb/C), the American Heart Association (35) (IIa/C), and the Society for Cardiovascular Angiography and Interventions (36) concerning the initialization of MCS (Table 1).

**TABLE 1:** Stages and management of cardiogenic shock based on the expert consensus of the Society for Cardiovascular Angiography and Interventions (36).

Stage	Description	Therapy
A At risk	Patients without current CS signs or symptoms but “at risk” of its emergence, including large ACS, non-STEMI, prior MI, and decompensated systolic or diastolic heart failure	Prepare for possible MCS need, ultrasound puncture of the femoral region
B Beginning CS	Pre-shock stage, clinical signs of relative hypotension or tachycardia without hypoperfusion	
C Classic CS	Patients with hypoperfusion requiring intervention to restore perfusion	Inotropes, vasopressors, and/or non-pVA-ECMO MCS required
D Doom/Deteriorating	Patients who remain unstable despite intense initial interventions, necessitating further escalation	pVA-ECMO needed
E Extremis	Patients experiencing circulatory collapse, often in refractory cardiac arrest, with ongoing CPR or concurrent support from multiple acute interventions, including ECMO	pVA-ECMO needed on-site, ECMO CPR may also be required

ACS, acute coronary syndrome; CPR, cardiopulmonary resuscitation; CS, cardiogenic shock; MCS, mechanical circulatory support; MI, myocardial infarction; STEMI, ST-elevation myocardial infarction; pVA-ECMO, peripheral veno-arterial extracorporeal membrane oxygenator.

### 3.2. Time management and eCPR

Currently, advancements in infrastructure, individual circumstances, financial considerations, and other factors have enabled the initiation of out-of-hospital pVA-ECMO augmented cardiopulmonary resuscitation (eCPR), possibly enhancing the prospects of long-term survival with favorable neurological outcomes for such patients (24, 37). These, however, impose an extreme burden on human resources and equipment allocation, yet robust data suggests a genuine increase in the survival of this patient population is not self-evident (26, 27, 38). A possible alternative option is the transportation of patients to a nearby tertiary shock center as soon as possible with maintained continuous advanced life support for precise and fast-paced pVA-ECMO implantation and initiation in a hospital setting (39, 40).

The present literature underscores the crucial role of immediate and effective CPR as a critical determinant in the feasibility of implementing VA-ECMO and ultimately, patient survival (41). Specifically, it is emphasized that the “no-flow period,” which refers to the interval from the onset of cardiac arrest to the initiation of CPR should be limited to 5 min, whereas each second without CPR, the prospects for favorable neurological recovery diminish significantly (10, 41). Research indicates that an acceptable neurological outcome reduces to below 2% after 20 min (42) and below 1% after 30 min of CPR (“low-flow period”) (43).

Regarding the recommended time frame for transitioning to eCPR, it is suggested that consideration for this intervention be made at the 21-min mark of continuous CPR efforts (44). Patients with a low-flow period exceeding 60 min are less likely to benefit from the implementation of VA-ECMO, and beyond 90 min there is limited expectation of a favorable outcome (45, 46). The Extracorporeal Life Support Organization (ELSO) recommends conducting a prompt evaluation of the feasibility of eCPR and aims to keep the elapsed time from cardiac arrest to ECMO initiation within 60 min (47).

Our CS ACS protocol also defines clear parameters in case of CPR need, paying particular attention to cases of out-of-hospital cardiac arrest (OHCA), accounting for transportation times and other delays (48) (see Figure 2).

**FIGURE 3:**
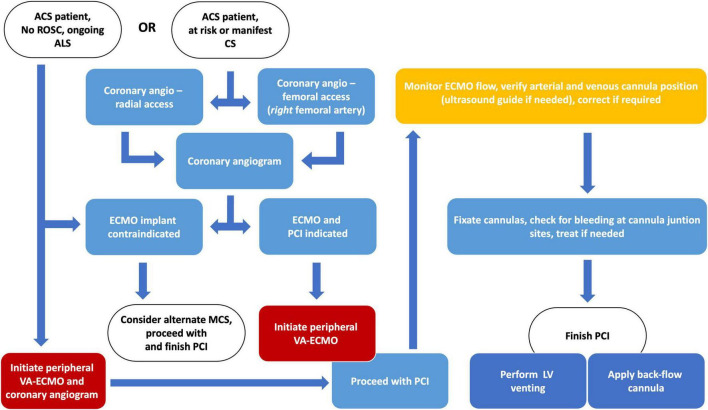
Algorithm and suggested workflow for the initiation of peripheral VA-ECMO in cardiogenic shock due to acute coronary syndrome. ACS, acute coronary syndrome; ALS, advanced life support; CS, cardiogenic shock; LV, left ventricle; MCS, mechanical circulatory support; PCI, percutaneous coronary intervention; ROSC, return of spontaneous circulation; VA-ECMO, veno-arterial extracorporeal membrane oxygenator.

The optimal timing of VA-ECMO implantation compared to PCI in CS ACS patients is a subject of considerable investigation. Research revealed that among this patient population, VA-ECMO initiation before revascularization results in significantly better short- and long-term outcomes compared to cases where VA-ECMO insertion occurs subsequent to revascularization (49–52). The recommended protocol from our center underscores the critical importance of prompt implementation of VA-ECMO before proceeding with revascularization procedures (see Figure 3).

## 4. Complications and management during pVA-ECMO care

### 4.1. Left ventricular unloading

In the case of femoral cannulation, the arterial cannula of the device, functioning as the outflow part, initiates a strong retrograde flow in the aorta, resulting in an elevation of left ventricular end-diastolic pressure (LVEDP) and increased wall tension, of an already failing heart in CS ACS. It may also increase myocardial oxygen demand and decrease the trans-coronary pressure gradient. This cascade of events may potentially lead to further myocardial necrosis, additionally compromising the unstable hemodynamic conditions and the function of a weak left ventricle (LV) (13, 53, 54). These factors can significantly contribute to unfavorable outcomes, especially in CS ACS where the sudden and significant reduction in cardiac function is primarily due to the imbalance in oxygen demand and supply. These processes are greatly influenced by the rate of ECMO flow, an increase of which can result in pulmonary stasis (so-called “ECMO lung”) and a decrease or even total cessation in cardiac output, potentially leading to left ventricular thrombosis, which harbors an extremely high mortality rate (55).

Based on the presented facts and observations, there is a clear indication of methods that can potentially reduce left ventricular pressure and congestion during VA-ECMO support. Two questions arise: what device to utilize and when should we implant it?

Although various methods are available for this purpose, decision-making can be challenging in the absence of clear guidelines. The two viable approaches are pharmacological therapy and the concomitant implantation of further invasive devices in conjunction with pVA-ECMO support (56).

**FIGURE 4:**
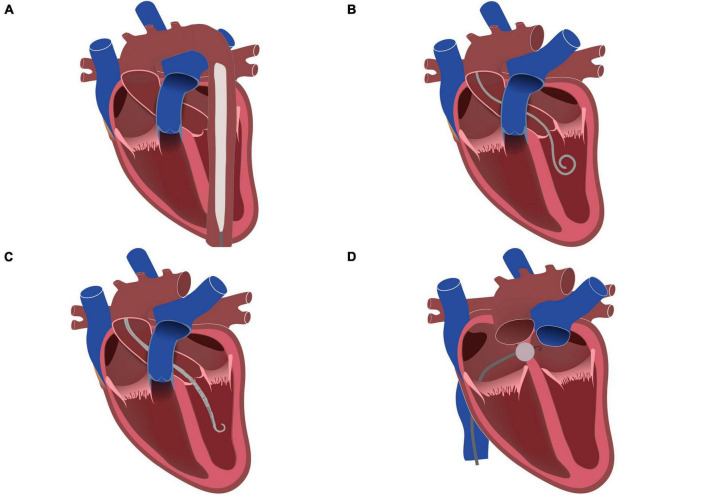
Left ventricular unloading methods during VA-ECMO treatment. **(A)** Intra-aortic balloon pump (IABP); **(B)** Pigtail catheter; **(C)** Microaxial transvalvular assist device (Impella); **(D)** Interatrial septostomy.

The former method primarily entails the administration of inotropes, which may be supplemented by diuretics or potentially continuous veno-venous hemofiltration (CVVH) to effectively decrease the intravascular fluid volume (10). The option of combined device utilization encompasses several techniques: initiation of intra-aortic balloon pump (IABP) counterpulsation to decrease left ventricular pressure and pulmonary edema by reducing the afterload (57) (I); percutaneous implantation of a pigtail catheter into the LV and connection of this unit to the pVA-ECMO venous cannula (58) (IIa); using a transaortic axial flow heart pump to enhance forward flow and reduce pulmonary capillary wedge pressure (59) (IIb); performing mechanical left ventricular decompression through a transseptal puncture and creation of iatrogenic atrial septal defect (ASD), resulting in a left-right shunt (60) (III) (see Figure 4).

The indication for left ventricular unloading in general, the preferred method, and the appropriate timing of unloading present critical, yet unsettled aspects. Hemodynamic monitoring plays a crucial role in comprehensive patient care and should encompass the examination of pulmonary arterial diastolic pressure and PCWP using a Swan–Ganz catheter (61). Furthermore, with the advancements in echocardiography, it became feasible to not only monitor the opening of the aortic valve and chamber dimensions but also to assess left ventricular function in greater detail using functional parameters like global longitudinal strain, determined by the preload- and afterload-dependent speckle-tracking echocardiography (62, 63). The findings should guide the decision in the indication, selection, and timing of the left ventricular unloading devices.

In a comparison between prophylactic (initiated simultaneously with VA-ECMO) and therapeutic (post-symptomatic) left ventricular unloading, Na et al. (64) found that the prophylactic approach resulted in reduced early mortality rates and higher success rates of bridging to cardiac replacement therapy. These findings were corroborated by a multicenter study, demonstrating that unloading initiated before or within 2 h of VA-ECMO implantation led to a lower 30-day mortality rate compared to ECMO alone. The research team also revealed that every hour spent without unloading increases the risk of mortality in this critically ill patient population (65, 66). These results emphasize the importance of prompt left ventricular unloading in improving patient outcomes.

### 4.2. Harlequin syndrome

Another phenomenon to account for that may occur during pVA-ECMO care is the mixing of ECLS oxygenated blood from the arterial cannula and the oxygen-deprived ejected blood from the LV in the proximal part of the descending aorta. Since CS ACS always involves some degree of pulmonary failure, blood exiting the left ventricle is left at low oxygen saturation levels. Mixing usually occurs at the site of the major supra-aortic branches, involving either one of these (brachiocephalic trunk, left carotid, and left subclavian artery). This is the phenomenon of differential hypoxemia, the so-called Harlequin or North-South Syndrome (10, 67), which may compromise effective arterial ECMO blood flow to the central nervous system, especially limiting oxygen-rich blood flow to the right carotid and vertebral arteries. Also, the upper, mainly right side of the body, especially the upper extremity may be affected causing severe ischemia on occasion. Yet, as in CS ACS left ventricular ejection is usually minimal or completely absent due to severe myocardial stunning, this phenomenon rarely occurs in the early stages of care. Later, during intensive care as LV function improves, this phenomenon may manifest and thus, must be monitored (10, 67).

Treatment of apparent Harlequin syndrome is urgent and involves potentially restoring pulmonary function to allow for physiological blood oxygenation and/or aggressive venting of the LV to decrease the volume of ejected oxygen-deprived blood. Additional methods involve converting to a V-AV system arrangement using a return cannula to the superior vena cava, transitioning to a jugulo-femoral V-A, and opting for a VV-ECMO configuration. A markedly invasive option to solve the problem is converting the peripheral ECLS system to a central configuration which allows for a near-physiological distribution of arterial blood (67–69).

### 4.3. Critical ischemia of the lower limb

Ipsilateral ischemia in the lower limb containing the arterial cannula of the device is a frequently observed manifestation. With the utilization of large-bore cannulas operating at high flow rates, there is an occurrence of flow obstruction to distal arterial segments, potentially leading to severe or even critical ischemia in the lower limb. Methods on how to manage this situation are not uniform, although the application of a back-flow cannula is the most recommended approach to address this issue (70, 71). It involves the ipsilateral anterograde puncture and sheath cannulation of the affected limb at a distal segment of the superficial femoral artery. This second sheath is afterward connected to the arterial cannula of the pVA-ECMO, restoring peripheral arterial circulation in the lower extremities. Similar to ventricular unloading, prophylactic and symptomatic back-flow cannula implementation methods may also be used (72).

### 4.4. Bleeding complications

Hemorrhagic events are one of the primary complications associated with VA-ECMO treatment, typically arising due to vascular cannulation, anticoagulation, and antiplatelet strategies, or perturbations induced in the coagulation cascade (9, 54).

Patients under ECLS support require comprehensive anticoagulation using unfractionated heparin (UFH), maintaining elevated activated clotting time levels (180 to 200 s) to prevent oxygenator and cannula clotting issues. Evaluation of activated partial thromboplastin time (aPTT) serves as a superior indicator to assess potential thrombotic events. These are to be adjusted to between 40 and 60 s and may be increased to the 60 to 80-s range in case of increased thrombotic risk (10, 73, 74). Newer literature emphasizes the concomitant measurement of anti-Xa factor in conjunction with aPTT for the possible best conjunction when administering UFH anticoagulation. Target levels for anti-Xa factors range from 0.3 to 0.7 IU/mL (75).

To further complicate bleeding issues percutaneous revascularization treatment of CS ACS is almost always indicated, and implantation of one or more coronary stents is performed. The existing evidence concerning the simultaneous use of antiplatelet and anticoagulant therapy in cardiogenic shock patients on VA-ECMO is notably limited (76). These require dual antiplatelet therapy for a certain period varying from days to months, often encompassing highly effective P2Y_12_ antagonist administration such as prasugrel of ticagrelor.

Therefore, significant external and internal (mainly gastrointestinal) bleeding complications may often occur in association with pVA-ECMO, requiring intensive care measures such as blood transfusions and adjustments to anticoagulant treatment (10).

Implanting large-bore cannulas entails the use of highly invasive techniques, often necessitating incisions at the insertion sites. Despite sutures being the common method for securing cannulas, there is currently no standardized approach for addressing para-sheath and para-cannula bleeding. According to the ELSO (77), securing peripheral cannulas must involve at least two sites to prevent malpositioning and decannulation. Ensuring effective fixation of the cannulas is essential due to the potential life-threatening complications it can avert, including ECMO cannula-related infections (CRI), as well as mechanical circuit dysfunction that can lead to air embolism or severe bleeding (78, 79).

When considering alternative sutureless securement devices (such as semi-occlusive dressings, modified tapes, and adhesive anchors), caution should be exercised, as these products are not specific for the purpose of ECMO cannula fixation and, therefore may not be recommended in this context without further evidence (79, 80).

## 5. Weaning from VA-ECMO

Standardized guidelines for weaning patients from pVA-ECMO are currently lacking. Usage is typically limited to a maximum of 21 days, mainly due to oxygenator failure, major bleeding, or limb ischemia, however, early weaning is recommended, whenever possible.

The decision to discontinue pVA-ECMO support should be based on objective clinical factors, and closely monitored indicators such as increasing blood pressure and decreasing central venous and pulmonary pressures. Acute de-cannulation in the catheterization laboratory, even if the clinical scenario is very hopeful, should be avoided. To assess true cardiac recovery and response during a weaning process, the utilization of trans-esophageal echocardiography (TEE) is recommended (81).

Weaning should be carried out gradually, following a standard reduction of flow rate by 1 l/h every 3–4 h (so-called ECMO challenge). Decannulation can be considered once the patient exhibits stability and is able to sustain adequate cardiovascular function without ECMO support for a minimum duration of 1–2 h. Methods of decannulation also vary from percutaneous closure to vascular surgery.

## 6. Our recommendations regarding pVA-ECMO use in the catheterization laboratory

### 6.1. Who requires pVA-ECMO support?

The clinical decision to implement pVA-ECMO support requires swift and decisive action. If an “at risk” or clinically manifest CS ACS patient is identified in the cath lab, the multidisciplinary Shock Team should be notified without hesitation (9, 15, 30). We recommend a delay of five to a maximum of 10 min from the initial identification of the patient to the first decision and reassessment of any prior decision by the Shock Team should there be a change in the clinical course of the patient. We strongly urge the Shock Team to develop and use an evidence-backed decision-making checklist, adjusted to hospital standards of care. This may be altered and perfected as the Shock Team gains experience and most importantly: it will negate the possibility of missing crucial inclusion or exclusion criteria and allow for a homogenized selection of potentially treatable patients (Figure 2).

Based on published recommendations (10, 47), our previous results and experiences (34), the most important inclusion criteria are genuine ACS clinical features (I), age of subject (II), and Killip presentation (III). Exclusion criteria deal with issues that either hinder effective pVA-ECMO function: severe peripheral artery and/or aortic disease, including but not limited to aortic dissection, surgically repaired aorta, iliac or femoral arteries (I), severe, acute bleeding (II), clinical presentation after systemic thrombolysis (III); or correspond to severe co-morbidities that render overall survival questionable, such as severe symptomatic chronic heart failure, significant valvular dysfunction, renal insufficiency or disseminated malignant disease (IV), and history of chronic alcohol, drug or other substance abuse (V).

It is important to note that patients who require ongoing or recurrent CPR demand further attention to detail from the Shock Team, to individually determine eligibility criteria. Here the main objective is to establish if the patient has any medical chance of survival in case of pVA-ECMO support initiation. We recommend focusing on clinical and time-based parameters to establish appropriateness. Sudden death occurring due to ventricular arrhythmias (I) with minimal no-circulation time, quick initiation of basic life support (II), and high exhaled CO_2_ levels (III) are important hallmarks of potential benefit from MCS. Furthermore, especially during ongoing advanced life support (ALS), transport time constraints play a crucial role, regarding the initiation of transport to the shock center (IV) and the transport time itself (V).

If the Shock Team deems the CS ACS subject eligible for pVA-ECMO support, initiation of MCS should be the priority after coronary anatomy assessment. With patients arriving undergoing continuous ALS, initiation before diagnostic coronary anatomy assessment should be carried out. If personnel exchange is required in the cath lab for pVA-ECMO implantation, it should be performed immediately. After the successful establishment of cannulation and flow support, subsequent care, often involving complex PCI, becomes considerably simplified, leading to improved potential outcomes (49–52).

### 6.2. How to implant the pVA-ECMO in the cath lab?

Our recommendation is that either a senior interventional cardiologist, or in specific cases, a qualified heart surgeon should perform implantation of the pVA-ECMO. The operator should be experienced with large bore (16–24F) catheter devices, ultrasound-based bi-femoral puncture techniques, and suture procedures. The summary of the proposed algorithm is illustrated in Figure 3.

#### 6.2.1. Puncture and cannulation

A bi-femoral cannulation method should be utilized in the cath lab during CS ACS. The right inguinal/femoral region is to be used for the venous cannula, ranging in size from 22 to 26F and lengths of up to 70 cm. The contralateral, left side is for the arterial cannula, with sizes between 14 to 18F (28, 29, 82). Our recommendation is to puncture the femoral vascular structures via ultrasound guidance *only*, ideally with one penetration of the needle. We propose maintaining this course of action even when cannulation is being performed during continuous chest compressions as part of ongoing ALS. Additionally, if patients are in the early stages of CS ACS (see Table 1), yet significant worsening is probable during the clinical course of PCI, pre-emptive cannulation of the femoral regions is preferable with 6F standard sheaths, which can either be efficiently exchanged to the pVA-ECMO cannula if needed or removed via commercially available closure devices (especially in case of the artery) if MCS is not required (36). These steps are essential for simplified and faster cannulation and are efficient in preventing later episodes of significant bleeding from puncture sites.

Cannula insertion is only advised using extra stiff or super stiff standard guidewires, using sequentially larger dilators, and finally the cannula itself. Based on our experience, it is often necessary to make a scalpel incision in the vessel before the final insertion of the cannula.

We also recommend placing pressure-resistant three-way valves on the arterial cannula side-port at this point during the procedure, which will serve as a connection point for the back-flow sheath. If left ventricular venting is planned via a pigtail catheter, then, the venous cannula side-port requires a similar valve as well. The distal end of the venous cannula needs to be placed into the right atrium (83), with the arterial cannula in the distal part of the abdominal aorta (28, 29, 82). Placement, depth, and positions of both cannulas need to be verified via fluoroscopy. If any uncertainties arise, positions may be checked and adjusted via ultrasound guidance (e.g., TEE for the venous side) (28, 29, 82). Connection of the pVA-ECMO circuit and cannulas requires the use of clamps and a syringe-based manual infusion of sterile saline into all four ends with meticulous attention to detail, to eliminate air bubbles from the system during connection, as any residuals can potentially cause catastrophic air emboli in the motor unit (84). Although unlikely to be swapped due to color coding, verifying arterial-arterial and venous-venous connections of the circuit and cannulas is mandatory as the last checkpoint prior to system launch.

#### 6.2.2. Cannula fixation and local bleeding management

After the system is correctly set up, the pVA-ECMO may be started after the removal of all clamps. Ideally, the puncture to pVA-ECMO start time should be below 15 min. After system startup, safely and effectively securing the cannulas is of paramount importance requiring at least three subsequent cutaneous sutures or multiple custom-made adhesive clasps (77). Para-cannula and para-sheath bleeding are commonly present and require attention. If bleeding from the access site is significant, we advise practitioners to not only suture the bleeding site but use commercially available potassium-ferrate-based hemostatic powders/paste or similar, specifically designed incised discs to cover the junction of the skin and cannula. We have found this method particularly effective in solving this problematic issue.

#### 6.2.3. Left ventricular unloading

Handling and fine-tuning the pVA-ECMO system should be left to dedicated personnel, ideally an expert perfusionist technician (77). With MCS stabilized circulatory and pulmonary support, revascularization should be completed as required in the CS ACS patient. Following successful PCI, there are two substantial matters to address concerning the pVA-ECMO system. The first one involves the decompression of the LV. Our standard of care mandates active LV venting for *every* CS ACS patient on pVA-ECMO support, regardless of the acute state of the chamber, or ejection fraction thereof. An additional ultrasound-guided arterial puncture of the right femoral region is required if not already present as PCI approach site. If accessible and available, the transaortic axial flow heart pump is the best option for active LV decompression as the device is adjustable to meet the clinical demands of the patient (59). The less sophisticated, non-adjustable alternative way with a similar mechanism of action involves a 7F pigtail catheter in the LV connected to the venous side of the pVA-ECMO cannula may also be recommended (58). We advise practitioners utilizing this technique to pay close attention to the venous cannula when attaching the catheter, as it can easily aspirate large amounts of air, rendering the motor unit and thus the whole MCS system useless. This is where the previously attached three-way valve plays a significant role, as it decreases the possibility of air emboli. A transseptal puncture to iatrogenic ASD formation is only recommended in case of a prosthetic aortic valve, it should not be undertaken otherwise (60). In a similar fashion use of the IABP for LV unloading is also contraindicated as it lacks the efficacy of active alternatives (57).

#### 6.2.4. Prophylactic back-flow cannula

As the final step of the procedure, the back-flow cannula should be implanted in the femoral artery distal to the arterial pVA-ECMO cannula (70, 71). We recommend applying this device to every patient, in a prophylactic manner. An ultrasound-based anterograde, precise puncture of the superficial femoral artery is required. We recommend using commercially available 7F metallic sheaths with reinforced ports, capable of handling the pressure of the arterial outflow cannula. Drawing from our practical knowledge, to facilitate connection, the three-way valve plays an important role in attaching the devices safely without compromising blood flow, or the danger of bleeding from the cannula side port.

### 6.3. Infrastructure requirement and training

To successfully initiate a pVA-ECMO-based MCS program for an institution aiming to take on shock center responsibilities, we recommend the following resources in terms of human resources: ECMO-trained senior interventional cardiologist or heart surgeon (I), ECMO-trained perfusionist (II), ECMO-trained interventional assistant (III), and anesthesiologist (IV). With respect to the infrastructure: a pVA-ECMO system *inside the cath lab 24/7* with an attached circuit, ready for the priming sequence. If the MCS device itself is outside of the cath lab, especially in a storage facility, this leads to further, significant time delays and reduces effectiveness. Our data shows that having the system inside the cath lab can halve puncture-to-ECMO start times.

Furthermore, an ECMO kit should be compiled encompassing everything that is needed in the cath lab for percutaneous implantation, fixation, and initiation of the system. We recommend including all consumables, e.g., the initial 6F sheaths, extra stiff guidewires, hemostatic devices, three-way valves, suturing and fixating materials, and all required clamps into the kit. Using this approach simplifies peri-procedural care.

Usually, that cath lab is a crowded environment, with most of the available space being taken up by the angiography system, its examination table, monitor and gantry. Also, other essential infrastructure is located in the examination room. As such, the available space is constrained, allowing only a few points of contact with the patient. Thus, it is advisable to pre-ordain the exact location of the pVA-ECMO machine, ensuring generous cable length compatibility and allocating applicable places for all medical staff. We support the idea of boot-camp-style training with a virtual patient (e.g., dummy) to focus on individual responsibilities, coordination, communication, and appropriate locations of staff and machinery.

### 6.4. Summary of recommendations

As a short summary of our recommendations, we advise always assessing the problem of CS ACS from a three-way perspective: patient (I), ECMO implant procedure (II), and infrastructure (III). These are three clearly definable, yet different issues when working as a Shock Team.

– Assessing the appropriate CS ACS patient for pVA-ECMO support needs to happen swiftly. The Shock Team must reach a clinically sound, unanimous verdict in a matter of minutes, for which a checklist is recommended to be used as discussed previously.
– The exact steps of the pVA-ECMO implementation need to be followed in a precise and calm manner for optimal performance. This is required to minimize puncture-to-ECMO time and prevent possibly catastrophic complications.
– The maintenance and replacement of the MCS system, other infrastructure and required consumables need to be undertaken at regular intervals and fully replenished after each implantation procedure.

Following the above recommendations and thought processes we hope that practitioners will be able to gain meaningful additional knowledge for their own Shock Team operations.

## Author contributions

RE: Writing–original draft. BN: Writing–review and editing. PK: Writing–review and editing. GF: Writing–review and editing. DB: Writing–review and editing. BK: Writing–review and editing. EZ: Writing–review and editing. BM: Writing–review and editing. IÉ: Writing–original draft.
